# Comparing factors affecting commencement and cessation of betel quid chewing behavior in Taiwanese adults

**DOI:** 10.1186/1471-2458-8-199

**Published:** 2008-06-05

**Authors:** Shue-Fang Yap, Pei-Shan Ho, Hsiao-Ching Kuo, Yi-Hsin Yang

**Affiliations:** 1 Faculty of Dental Hygiene, College of Dental Medicine, Kaohsiung Medical University, Taiwan; 2 Statistical Analysis Laboratory, Division of Clinical Research, Kaohsiung Medical University Chung-Ho Memorial Hospital, Kaohsiung Medical University, Taiwan

## Abstract

**Background:**

Betel quid is the fourth most common used substance in the world after tobacco, alcohol and caffeine. Although factors related to betel quid chewing or cessation of behaviors were reported previously, few studies simultaneously compared both behaviors in the same population. In addition, it is essential to consider time-to-event concept, since the chance of developing or stopping habit may vary over time. The purpose of this study was to compare the risk factors for commencement and cessation of betel quid chewing behaviors in a time-to-event setting.

**Methods:**

A stratified multi-stage cluster sampling with selection probabilities proportional to size (PPS) was designed for Taiwanese adults with aged 18 years old and above. Kaplan-Meier estimates and Cox proportional hazard regression models were used to compare and calculate the hazard rate ratios for related factors to commencement or cessation of chewing habits.

**Results:**

In Taiwan, men had a higher betel quid chewing rate (M: 20.9%, W: 1.2%), but woman chewers had a lower cessation rate (M: 27.5%, W: 12.7%). The hazard rate ratio (HRR) of having chewing habit changed from 4.22 (men vs women) univariately to 1.38 multivariablely, which indicated gender differences were confounded by other factors. In multivariable analysis, the risk factors of gender, education and ethnicity were significantly associated with both starting and cessation of betel quid chewing behavior. The factors of occupation, cigarette smoking and alcohol drinking were only associated with starting habit.

**Conclusion:**

Commencement or cessation of chewing behavior involves a scenario of time, hence it is preferable to use a time-to-event approach for the comparison. The cessation rates of betel quid chewing were decreasingly associated with the daily consumption of betel quid. Hence, reducing of daily amount in betel quid cessation program may be associated with future stopping habit.

## Background

Betel quid is the fourth most universally used substance in the world after tobacco, alcohol and caffeine [1]. It is chewed periodically by at least 10% of the worlds population (600 million persons) globally [1], and it is estimated that 10% of the population (2 million persons) in Taiwan chew betel quid [2]. In several south, Southeast Asian and Asia Pacific communities, betel quid is chewed for many reasons, including for its psychostimulating effect, as well as a social and cultural practice [3]. Scientific evidence has shown that chewing betel quid contributes independently to the risk of oral cancer, oral mucosal lesions, oral leukoplakia and oral submucous fibrosis [3]. These relevant health risks emphasize the necessity for betel quid cessation. Betel quid chewing is a serious public health issue in Taiwan, and an effective strategy is necessary to implement betel quid cessation [3].

Commencement and cessation of chewing behavior has been discussed in numerous studies. However, there has been limited research to investigate both behaviors in the same study population. The purpose of this study was to use a community survey database to compare the risk factors associated with betel quid chewing, and cessation behavior. In addition, we investigated for the most commonly seen gender differences and, whether they are confounded by other effects.

## Methods

### Study population and survey sampling design

A stratified multi-stage cluster sampling with selection probabilities proportional to size (PPS) was designed to conduct and collect study participants. The study participants were residents of Taiwan aged 18 years old and above. Residents from the military, hospitals, rehabilitation institutes, schools, dormitories and correctional facilities were excluded from the sampling frame. In this survey, the stratification factors included geographical area, gender and age groups. The whole of Taiwan was divided into 26 strata, which included Taipei City, Kaohsiung City, 21 cities/counties, and 3 aboriginal areas. Within each geographical stratum, there were 8 strata, 2 gender groups (male and female) versus 4 age groups (18–34 years old, 35–49 years old, 50–64 years old and 65 years old or older).

The sample size for each geographical area was 400 (the actual number was 385 rounded to the nearest hundred). This number was determined by allowing 95% confidence intervals within 3% of the range for a prevalence of 10%. For some areas where the prevalence rates were from 1% to 50%, their 95% confidence interval was maintained within ± 1% to ± 5%. In order to maintain a large enough sample size for statistical power within each area/sex/age strata, a sample size of 400 within each area strata was equally divided into 8 gender/age strata. The estimated mean was then weighted according to the proportion of age/gender. The household registry of 2001 was used as the sampling frame.

### Questionnaire interview

Public health workers were trained to conduct personal interviews using a standard structured questionnaire. The questionnaire included demographic information, and details of betel quid chewing habit (average daily amount, type of quid, age of initial betel quid chewing, and age of cessation). A lifetime betel quid chewer was defined as a person who chewed at least one quid within a week for at least 6 months. Current betel quid chewers were those who currently have the chewing habit. Former betel quid chewers were defined as a person who had stopped the chewing habit for at least 6 months. There are three types of quid in Taiwan, betel quid is made with an unripe areca fruit and slaked lime paste with a piece of betel leaf. Lao-hwa quid is made with a piece of inflorescence of *Piper Betle Linn*, and red lime paste (slaked lime and some local flavoring) placed into an unripe areca fruit. Stem quid is made with the stem of *Piper Betle Linn *into an unripe areca fruit.

### Statistical analysis

The estimated population proportions and mean were weighted according to the population structure. There were a total of 208 strata (26 areas × 8 sex/age = 208). The population size of age 18 years old or older in 2001 was first obtained for each strata (N_i_, i = 1 to 208). Let the sample size of each strata be n_i _(i = 1 to 208), then the weight for each strata can be obtained by ${\text{W}}_{\text{i}}=\frac{N_{i}}{n_{i}}•\frac{n}{N}$, where $\text{N}=\sum_{i=1}^{208}N_{i}$, $\text{n}=\sum_{i=1}^{208}n_{i}$ and the "i" is one of the area/sex/age strata. SUDAAN software was used to implement the statistical computation for proportions and 95% confidence intervals were used to show the precision of estimates. In addition, the relation between socio-demographic characteristics and lifestyle habits were computed by chi-square test.

To investigate the commencement and cessation of betel quid chewing behavior in terms of time (age in years), the Kaplan-Meier estimate was used to calculate the hazards rate for life time betel quid chewers and former chewers. There were two events in this study. The first event is a person who started betel quid chewing behavior. The follow-up period for the time-to-event was length of time between birth to the age of starting betel quid chewing behavior. The second event is the person who had stopped chewing behavior. The follow-up for the time-to-event were length of time between ages of start chewing behavior to the age of stop betel quid chewing. The log-rank test was used to compare the difference among the groups. Furthermore, the Cox proportional hazard regression model was used to calculate the hazard rate for factors related to commencement or cessation of chewing habits. P-values lower than 0.05 were considered statistically significant. These analyses were conducted by the Statistical Analysis System (SAS Inc.).

## Results

There were 11,723 participants in this survey which 54 participants without complete information on chewing habit were excluded from the analysis. The prevalence rates of betel quid chewing between men and women in different demographic characteristics are shown in Table 1. The results showed that males (20.9%) had a higher betel quid chewing rate than females (1.2%). In each variable, males had a higher betel quid chewing rate than females. In terms of age, males aged 35–49 years old (23.9%) had the highest chewing rate, while females aged 65 years old and above (2.3%) had the highest rate. In regards to education level, males with junior high school level and females with primary school level were the highest categories among the betel quid chewers (32.1% and 2.8%, respectively). For both genders, persons with college education and above had the lowest proportion of chewers. The highest betel quid chewing for occupation were technical (males 29.8% and females 1.5%). In an aboriginal population the highest chewing prevalence were in both genders. Moreover, more cigarette smokers and alcohol drinkers also had chewing behavior.

**Table 1:** Prevalence of betel quid chewing behavior between men and women for different demographics characteristics

Variables	Item	Betel quid chewers							
		
		Male				Female			
			
		Adjusted sample size	%	95% Confidence Interval	P-value	Adjusted sample size	%	95% Confidence Interval	P-value
Total		5922	20.9	(19.2, 22.6)		5747	1.2	(1.0, 1.6)	
									
Age group	18–34	2682	21.0	(18.1, 24.2)	<.0001	2597	0.6	(0.4, 1.1)	0.0017
	35–49	1790	23.9	(21.0, 27.0)		1769	1.6	(1.0, 2.6)	
	50–64	919	20.8	(18.5, 23.3)		925	1.7	(1.2, 2.3)	
	65 and above	531	10.4	(8.9, 12.2)		456	2.3	(1.7, 3.1)	
									
Education #	Literate	283	19.0	(15.3, 23.3)	<.0001	653	2.2	(1.7, 2.9)	<.0001
	Primary school	921	26.4	(23.3, 29.7)		1153	2.8	(2.1, 3.6)	
	Junior high school	1021	32.1	(27.6, 36.9)		802	1.1	(0.6, 1.9)	
	Senior high school	2100	25.6	(22.3, 29.3)		1775	0.9	(0.4, 2.1)	
	College and above	1571	4.6	(3.2, 6.5)		1345	0.0	(0.0, 0.1)	
									
Occupation #	Unemployed or non-technical	2903	19.5	(17.3, 21.9)	<.0001	3893	1.4	(1.1, 1.8)	0.0731
	Technical	1488	29.8	(26.4, 33.4)		649	1.5	(1.0, 2.2)	
	Semi-professional	865	18.2	(13.8, 23.7)		895	0.7	(0.1, 3.6)	
	Managerial/professional and above	561	7.4	(4.1, 13.1)		310	0.0	(0.0, 0.1)	
									
Ethnicity #	Aborigines	111	54.3	(44.9, 63.5)	<.0001	106	33.8	(28.1, 39.9)	<.0001
	Non-aborigines	5797	20.2	(18.6, 22.0)		5631	0.6	(0.4, 1.0)	
									
Cigarette smoker #	Yes	2979	37.1	(34.2, 40.1)	<.0001	190	14.8	(8.6, 24.1)	<.0001
	No	2918	4.4	(3.5, 5.5)		5553	0.8	(0.6, 0.9)	
									
Alcohol drinker	Yes	815	51.7	(46.3, 57.2)	<.0001	60	21.0	(14.5, 29.4)	<.0001
	No	5107	16.0	(14.4, 17.7)		5687	1.0	(0.8, 1.4)	

#: 26 males and 19 females with missing value in education; 105 males with missing value in occupation; 14 males and 10 females with missing value in ethnicity; 25 males and 4 females with missing value in cigarette smoker

For people with a lifetime chewing habit, the proportion of those who stopped the chewing habit for at least 6 months is shown in Table 2. Females had a lower cessation rate than males (12.7% and 27.5%, respectively). The proportion of those who quit the chewing habit was statistically significantly different in age group (p < .0001), education (p = 0.0003), ethnicity (p = 0.0004), cigarette smoking (p = 0.0113) and type of quid (p < .0001) in males. However, none of the factors were statistically significantly different in females. In terms of age groups, more elderly people (age 65 years old and above) quit the chewing habit (males 50.5%, females 22.1%). In all categories fewer females quit the chewing habit than males.

**Table 2:** Proportion of chewers stopping chewing habit between men and women in different demographic characteristics

Variable	Item	% of stop chewing habit							
		
		Male				Female			
			
		Adjusted sample size	%	95% Confidence Interval	P-value	Adjusted sample size	%	95% Confidence Interval	P-value
Total		1237	27.5	(23.8, 31.6)		71	12.7	(7.5, 20.8)	
									
Age group	18–34	563	21.9	(15.7, 29.7)	<.0001	17	4.3	(1.4, 12.2)	0.5351
	35–49	428	26.4	(21.1, 32.5)		29	11.7	(3.9, 30.5)	
	50–64	191	40.0	(33.8, 46.6)		16	17.3	(7.4, 35.5)	
	65 and above	55	50.5	(41.9, 59.1)		9	22.1	(11.2, 38.8)	
									
Education #	Literate	54	42.5	(31.4, 54.3)	0.0003	14	16.7	(7.8, 32.3)	0.6409
	Primary school	243	35.1	(28.9, 41.7)		32	17.1	(8.3, 32.0)	
	Junior high school	328	22.6	(16.5, 30.1)		9	10.3	(3.1, 28.8)	
	Senior high school	538	24.8	(18.5, 32.3)		16	1.9	(0.4, 8.7)	
	College and above	72	35.2	(19.2, 55.4)		0	0.0	(-, -)	
									
Occupation	Unemployed or non-technical	564	30.8	(25.2, 37.0)	0.1301	55	16.4	(9.8, 26.1)	0.4067
	Technical	473	24.5	(19.3, 30.7)		10	0.7	(0.1, 4.9)	
	Semi-professional	158	25.0	(14.2, 40.2)		6	0.0	(-, -)	
	Managerial/professional and above	42	27.2	(9.6, 56.9)		0	0.0	(-, -)	
									
Ethnicity #	Aborigines	60	7.8	(5.1, 11.9)	0.0004	36	9.8	(4.6, 19.7)	0.4538
	Non-aborigines	1173	28.6	(24.7, 32.9)		35	15.7	(7.3, 30.6)	
									
Cigarette smoker #	Yes	1105	26.5	(22.6, 30.8)	0.0113	28	7.2	(3.2, 15.7)	0.2572
	No	128	37.1	(26.1, 49.6)		43	16.4	(9.0, 28.0)	
									
Alcohol drinker	Yes	422	27.9	(21.1, 35.9)	0.8412	13	5.9	(2.5, 13.2)	0.4259
	No	815	27.4	(23.1, 32.1)		58	14.2	(8.0, 24.0)	
									
Type of quid #	Betel quid	786	20.3	(16.2, 25.0)	<.0001	55	13.1	(7.2, 22.7)	0.8280
	Lao-hwa quid	259	44.1	(35.5, 53.1)		2	0.0	(-, -)	
	Betel quid + Lao-hwa quid	134	31.0	(19.0, 46.3)		6	6.4	(0.8, 37.3)	
	Others	50	47.9	(27.1, 69.4)		8	19.3	(6.6, 44.7)	

#: 2 males with missing value in education; 4 males with missing value in ethnicity; 4 males with missing value in cigarette smoker; 8 males with missing value in type of quid

**Figure 1:**
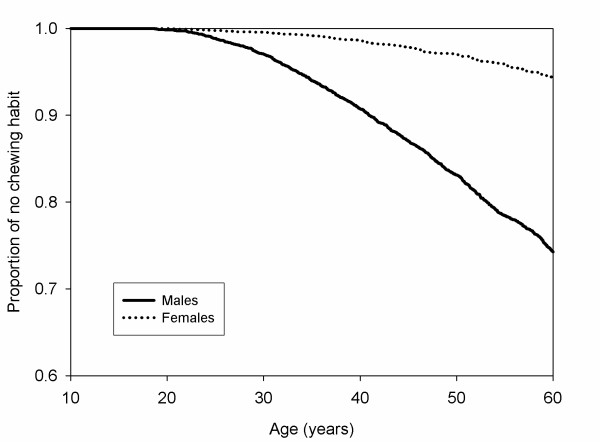
From birth to commencement of betel quid chewing between males and females.

**Figure 2:**
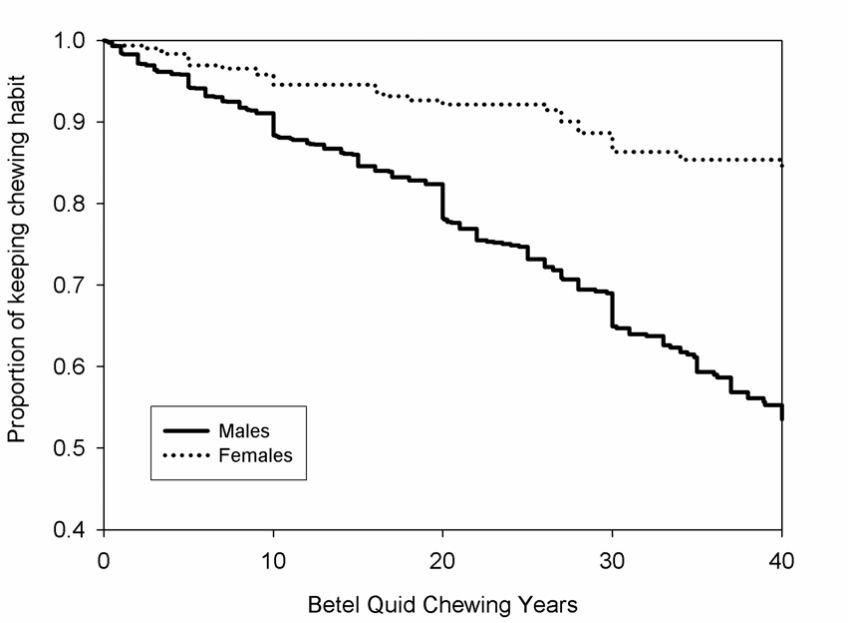
**Commencement to cessation of betel quid chewing between males and females.** Refer to main text for details.

To estimate the time (age in years) to commencement of the chewing habit, and the time (age in years) to cessation of the chewing habit between males and females, the Kaplan-Meier estimates are computed and plotted in Figure 1 and 2. The curve for the age of women starting the habit was above the curve of men, which indicated that women were less likely to develop the chewing habit. The chewers began chewing betel quid at 16 years old. Figure 2 shows that the curve for the age of women stopping the chewing habit was above the curve of men, this showed that women are less likely to stop betel quid chewing. Both lines were approximately linear, which indicates that there were no peak time intervals for developing or stopping the chewing habit. Therefore, cessation of the chewing habit happens all the time in both genders. Log-rank tests indicated that the p-values were significant (p < 0.0001) for both betel quid chewing and betel quid cessation.

To evaluate the factors related to stopping the betel quid chewing habit between men and women in terms of time, the hazard rate had estimates by univariate and multivariable Cox-regression as shown in Table 3. When considering developing chewing habits, in univariate analysis most factors were statistically significant, and for men (HR = 4.22, 95% CI = 3.74–4.77, p < .0001), education of junior high school (HR = 2.25, 95% CI = 2.00–2.54, p < .0001), senior high school (HR = 2.58, 95% CI = 2.31–2.89, p < .0001), technical (HR = 3.12, 95% CI = 2.81–3.47, p < .0001), Taiwan aborigines (HR = 4.41, 95% CI = 4.00, 4.87, p < .0001), smokers (HR = 7.04, 95% CI = 6.32–7.85, p < .0001) and drinkers (HR = 4.46, 95% CI = 4.04–4.92, p < .0001) all had higher risk (hazard rate). However, in multivariable Cox-regression the risk of men starting chewing behavior became lower (HR = 1.38, 95%CI = 1.20–1.59, p < .0001). Hence, the phenomenon of more males developing the habit may be affected by others factors in the model.

**Table 3:** Univariate and multivariable analysis of betel quid from birth until commencement of chewing betel quid

Variables	Item	Univariate			Multivariable		
			
		Hazard Rate Ratio	95% Confidence Interval	P-value	Hazard Rate Ratio	95% Confidence Interval	P-value
Sex	Male	4.22	(3.74, 4.77)	<.0001	1.38	(1.20, 1.59)	<.0001
	Female	1.00			1.00		
							
Education	Literate	0.30	(0.26, 0.34)	<.0001	0.47	(0.37, 0.61)	<.0001
	Primary school	1.01	(0.92, 1.12)	0.8400	0.92	(0.73, 1.17)	0.4969
	Junior high school	2.25	(2.00, 2.54)	<.0001	2.33	(1.83, 2.97)	<.0001
	Senior high school	2.58	(2.31, 2.89)	<.0001	2.51	(1.99, 3.17)	<.0001
	College and above	1.00			1.00		
							
Occupation	Unemployed or non-technical	0.35	(0.31, 0.38)	<.0001	0.97	(0.69, 1.37)	0.8800
	Technical	3.12	(2.81, 3.47)	<.0001	2.09	(1.48, 2.96)	<.0001
	Semi-professional	1.73	(1.44, 2.09)	<.0001	1.52	(1.04, 2.20)	0.0288
	Managerial/professional and above	1.00			1.00		
							
Ethnicity	Aborigines	4.41	(4.00, 4.87)	<.0001	4.38	(3.94, 4.87)	<.0001
	Non-aborigines	1.00			1.00		
							
Cigarette smoker	Yes	7.04	(6.32, 7.85)	<.0001	4.10	(3.62, 4.63)	<.0001
	No	1.00			1.00		
							
Alcohol drinker	Yes	4.46	(4.04, 4.92)	<.0001	1.73	(1.56, 1.92)	<.0001
	No	1.00			1.00		

When considering years to stop chewing behavior, men were more likely to stop chewing than women in multivariable analysis was shown in Table 4 (HR = 1.73, 95%CI = 1.14–2.64, p = 0.0107). The most likely subgroup of stopping chewing behavior were men, college and above education, semi-professional occupation, non-aborigines, non-smokers, non-drinkers, type of quid and daily betel quid chewing number. Furthermore, daily betel quid chewing consumption of 30 pieces or more (HR = 0.69, 95%CI = 0.52–0.90, p = 0.0070), and 100 pieces or more (HR = 0.57, 95%CI = 0.30–1.07, p = 0.0785) were significantly less likely to stop chewing in multivariable analysis. Lao-hwa quid was the highest cessation of chewing type of quid in our study, they were 1.71 times more likely to stop chewing. In addition, those who chew a mixture of betel quid and Lao-hwa quid were 1.70 times more likely to stop chewing behavior.

**Table 4:** Univariate and multivariable analysis of betel quid chewer to quit betel quid

Variable	Item	Univariate			Multivariable		
			
		Hazard Rate Ratio	95% Confidence Interval	P-value	Hazard Rate Ratio	95% Confidence Interval	P-value
Sex	Male	3.48	(2.44, 4.97)	<.0001	1.73	(1.14, 2.64)	0.0107
	Female	1.00			1.00		
							
Education	Literate	0.54	(0.41, 0.70)	<.0001	0.46	(0.28, 0.76)	0.0026
	Primary school	0.95	(0.77, 1.16)	0.5986	0.59	(0.37, 0.95)	0.0294
	Junior high school	1.23	(0.94, 1.62)	0.1281	0.67	(0.40, 1.11)	0.1156
	Senior high school	1.62	(1.26, 2.09)	0.0002	0.74	(0.46, 1.21)	0.2309
	College and above	1.00			1.00		
							
Occupation	Unemployed or non-technical	0.76	(0.62, 0.93)	0.0083	1.19	(0.59, 2.41)	0.6295
	Technical	1.14	(0.92, 1.43)	0.2301	1.17	(0.58, 2.39)	0.6584
	Semi-professional	1.71	(1.17, 2.50)	0.0058	1.46	(0.68, 3.12)	0.3315
	Managerial/professional and above	1.00			1.00		
							
Ethnicity	Aborigines	0.29	(0.22, 0.37)	<.0001	0.40	(0.30, 0.55)	<.0001
	Non-aborigines	1.00			1.00		
Cigarette smoker	Yes	1.64	(1.28, 2.09)	<.0001	0.95	(0.73, 1.25)	0.7261
	No	1.00			1.00		
							
Alcohol drinker	Yes	0.78	(0.63, 0.96)	0.0204	0.81	(0.65, 1.02)	0.0679
	No	1.00			1.00		
							
Type of quid	Betel quid	0.48	(0.39, 0.58)	<.0001	0.73	(0.50, 1.08)	0.1127
	Lao-hwa quid	2.63	(2.13, 3.25)	<.0001	1.71	(1.13, 2.58)	0.0104
	Betel quid + Lao-hwa quid	2.08	(1.48, 2.94)	<.0001	1.70	(1.04, 2.79)	0.0362
	Others	1.00			1.00		
							
Daily betel quid chewing amount (pieces)	1–9 pieces	1.00			1.00		
	10–19 pieces	0.99	(0.78, 1.26)	0.9385	0.81	(0.62, 1.06)	0.1276
	20–29 pieces	0.98	(0.77, 1.26)	0.8960	0.75	(0.56, 0.99)	0.0401
	30–99 pieces	0.87	(0.68, 1.10)	0.2489	0.69	(0.52, 0.90)	0.0070
	above 100 pieces	0.66	(0.36, 1.20)	0.1742	0.57	(0.30, 1.07)	0.0785

## Discussion

Many studies have discussed the factors relating to commencement of betel quid chewing as well as cessation of chewing behavior. Few studies parallelly investigated both behaviors at the same time. In this study, we used a community survey to identify possible reasons for both starting and stopping behavior.

Social expectations are different between genders in many societies, it may result in different chewing behavior among men and women. The lifetime chewing prevalence was found to be relatively low in women (1.2%) as compared to men (20.9%) in our study. Similarly, in India (men 34.5%, women 27.2%) [4], the Solomon Islands (men 83%, women 68%) [5] as well as current chewing prevalence in Taiwan (men 14.4%, women 1.5%) [6] also had a higher prevalence in men. Nevertheless, there are also societies where more women chew betel quid than men; such as Cambodia (men 3.2%, women 8.2%) [7], Pakistan (men 6.8%, women 40.6%) [8], indigenous people of Sarawak, Malaysia (men 30%, women 63%) [9] and Taiwan aborigines (men 60.6%, women 78.7%) [10]. Even so, in Thailand (men 16%, women 19%) [11] and Xiangtang City, China (men 39.3%, women 30.5%) [12] have more equal betel quid chewing prevalence between genders. The differences among men and women may be due to various reasons. When men chewed more than women, most of the time women tried to avoid red-stained lips, and foul smelling breath associated with betel chewing [4]. In contrast, men chewers may develop the chewing behavior to project an image of machismo, and strength [13]. The reasons for women having a higher chewing prevalence than men are mostly for traditional cultural factors [10,13].

The fact that men are less or more likely to chew betel quid has been reported in many studies. Whether gender differences fundamentally exist or are confounded by other factors as mentioned above is of interest. In our study, males were 4.22 times more likely to have chewing behavior in the univariate analysis, but were only 1.38 times more likely to have chewing behavior, after adjusting for education, occupation, ethnicity, smoking and drinking habits. It may imply that the tendency of men being more likely to chew is affected by other factors. In other words, men are more likely to chew betel quid than women due to different combinations of factors such as education, occupation, ethnicity, cigarette smoking and alcohol drinking between genders.

Those in a technical occupation were more likely to chew betel quid (HR = 2.09) similar to a previous study in Taiwan [14]. Winstock reported that areca nut contains arecoline, a para-sympathomimetric agent, and can stimulate salivation and sweating [15]. Therefore, blue collar workers are more likely to use betel quid for its physical effects, a sense of well-being, heightened alertness, reduction of tension, and hence increasing capacity to work [16,17]. In addition, they might also be influenced by the social environment, and long working hours, with betel quid being perceived as a stimulant [15,18]. An economic reason may also play a part in the habit differences. In Taiwan, betel/areca quid are mostly sold at betel stands except for aboriginal communities. Twenty pieces of betel quid generally costs NT$100 (US$3.20), while a pack of cigarette (20 cigarettes) only costs NT$35 (US$1.10) on average. Therefore, the unemployed or those on a lower income may not be able to afford the betel quid chewing habit.

Betel quid plays an important role in Taiwan aborigines cultural activities who have a long history of chewing habits [10]. This is similar to many Southeast Asian countries and India, which also have a long history of chewing betel quid, and a cultural and social acceptance of the practice [11]. That is the reason for a high chewing prevalence among Taiwanese aborigines.

Betel quid chewing behavior in different countries may be affected by different cultural and socio-demographic factors. Cambodia has the highest chewing habit among the older population [19]. In the Solomon Islands, education level and cigarette smoking habit are associated with betel quid chewing behavior [5]. Previous studies in Taiwan have shown that gender, education level, occupation, ethnicity, cigarette smoking and alcohol drinking are associated with betel quid chewing [6,20].

Betel quid prevention has become an important health issue recently. It is related to oral disease and other health problems, such as asthma [21], cardiovascular disease [22], diabetes mellitus [23,24], periodontal disease [25], oral submucous fibrosis [16,26], and oral cancer [25]. Hence these act as an important factor in betel quid prevention and cessation strategies to reduce oral disease.

Our findings indicate that though betel quid chewing is more common among male chewers, but female chewers are less likely to stop chewing betel quid (male 27.5%, female 12.7%). Previous studies in Taiwan also showed that prevalence of betel quid cessation (males 12.8%, females 0.9% [27], males 45%, females 0.2% [6], and Taiwan aboriginal males 8.2%, females 6.7% [20]) were lower among the women chewers. In our study, men are 1.73-fold more likely than women to stop chewing.

In general, previous studies showed that education level, occupation, alcohol drinking, and cigarette smoking are related to cessation of betel quid chewing [6,18,20]. In addition, our study showed that gender, ethnicity and type of quid are significant factors associated with betel quid cessation behavior among chewers in terms of time. Women had a lower betel quid chewing rate and cessation of chewing rate than men in terms of time. Moreover, the time-to-event curves were linearly decreased over time, which indicated that cessation of the chewing habit happens all the time in both genders. The older population had the highest betel quid chewing cessation prevalence, which may be due to health problems. A recent study also showed that Taiwan aborigines (aOR = 0.40, 95%CI = 0.24–0.68) were less likely to stop chewing than non-Taiwan aborigines [20], similar to our study. It may be related to culture and tradition among the aborigines [10], thus the betel quid cessation is lower in this population.

In our findings, chewers with high daily consumption were less likely to stop chewing behavior. A recent study pointed out that the fewer pieces of betel quid chewed the more likely they were to stop chewing behavior which was similar to our finding (but not significantly) [20]. Betel quid was the most popular type of quid in this study. Nevertheless people who chew Lao-hwa quid had the highest rate of cessation (HR = 1.71). Moreover, those who chew mixtures of betel quid and Lao-hwa quid are 1.70 times more likely to stop chewing behavior. Lao-hwa quid is commonly seen in urban areas, it is made with a piece of inflorescence of *Piper Betle Linn*, and red lime paste (slaked lime and some local flavoring) into an unripe areca fruit [10]. Betel quid generally contains more slaked lime than Lao-hwa quid. Hence, the flavor is stronger than Lao-hwa quid. Therefore, it is less likely for betel quid chewers to stop chewing behavior.

## Conclusion

Commencement or cessation of chewing betel quid behavior involves a scenario of time-to-event, hence it is preferable to consider the analysis in terms of time (years to events). In this study, we found that women had a lower chewing behavior rate, but they were less likely to stop chewing than men. The factors related to the betel quid chewing habit and cessation is not parallel. This phenomenon has an impact on the social background of the chewers, such as occupation, education, ethnicity, cigarette smoking and alcohol drinking. As regard to whether betel quid chewing cessation has an impact on gender, ethnicity and type of quid, male chewer's serve as the major focus in betel quid chewing prevention programs. The cessation rates of betel quid chewing were decreasingly associated with the daily consumption of betel quid. Hence, reducing of daily amount in betel quid cessation program may be associated with future stopping habit.

## Competing interests

The authors declare that they have no competing interests.

## Authors' contributions

S–FY carried out the studies, participated in the sequence alignment, performed the statistical analysis and drafted the manuscript. P–SH participated in the design of the study. H–CK performed the statistical analysis. Y–HY led this study, and participated in its design and coordination and helped to draft the manuscript. All authors read and approved the manuscript.

## Pre-publication history

The pre-publication history for this paper can be accessed here:


